# Histology and morphometry of the skin of the trident goby *Tridentiger brevispinis* (Perciformes, Gobiidae)

**DOI:** 10.1186/s42649-022-00077-y

**Published:** 2022-08-09

**Authors:** Hyun-Tae Kim

**Affiliations:** Department of Science Education, Jeonju National University of Education, Jeonju, 55101 Republic of Korea

**Keywords:** Cutaneous respiration, Blood capillary, Dermal vascularization, Freshwater goby, Reduced diffusion distance

## Abstract

The Korean trident goby, *Tridentiger brevispinis*, lives in adverse habitats that can easily become hypoxic due to low precipitation, regional dry periods, and high amounts of solar radiation. Histological and morphometric studies revealed the goby’s specialized skin (35.4–150.0 μm in thickness), consisting of an epidermis and dermis. The thicker epidermis comprises an outermost surface layer (having taste buds, stratified flattened cells, mucous cells, pigment cells, and stratified polygonal cells), middle layer (having stratified polygonal cells), and stratum germinativum (stratified columnar cells). In particular, the dermis has scales, well-developed vascularization, and a few blood capillaries just above the basement membrane, and a reduced diffusion distance was present in the lateral body. Consequently, adaptations such as thicker epidermis, well-developed vascularization, few blood capillaries, and a reduced diffusion distance may provide cutaneous respiration for survival in poorly oxygenated water during the periodic dry season.

## Introduction

Teleosts have a respiratory system that allows for gas exchange of dissolved oxygen absorption and carbon dioxide emission between their body membrane and water (Fernandes, 2016). This physiological metabolism is observed in diverse internal organs such as gills (Lefevre et al. 2011; Blank and Burggren 2014), gastrointestinal surface (Grosell et al. 2010), swim bladder (Fernandes et al. 2012), branchial chamber (Sundin et al. 1999), labyrinthine organ (Zaccone et al. 2019), and skin (Glover et al. 2013). Among them, the skin is responsible for 5 to 30% of oxygen absorption in underwater teleosts (Graham 1997) that inhabit shallow and stagnant water with lower oxygen levels (Wright 2021). In addition, the skin obtains up to 50% percent of supplemental oxygen in amphibious fishes (Graham 2011) that are able to move in and out of the water (Ishimatsu 2017). For this physiological capability, there are specific skin histological characteristics: 1) intraepidermal blood capillaries developed in various positions of the epidermis, 2) well-developed vascularization along dermal collagen fibers, 3) prominent swollen cells, mucous cells, and club cells, and 4) reduced or absent scales (Kim and Park 2011; Glover et al. 2013).

The trident goby *Tridentiger brevispinis* prefers to live in slow-flowing streams or reservoirs with a rock, gravel, or pebble bottom, and is distributed along Korean peninsula, Japan, the Kuril Islands, and Sakhalin (Pietsch et al. 2001). This goby’s habitat undergoes extreme changes in water level due to dry and rainy seasonal patterns on the Korean peninsula (Kim et al. 2011), and may become a periodically slow-flowing water region with low dissolved oxygen content; stagnant water pools are created frequently throughout this aquatic environment. To overcome adverse hypoxic conditions during the dry season, many freshwater fishes have histological adaptations of the skin with blood capillary, epidermal, or dermal modifications (Park, 2002a, b; Park et al. 2003a; Park and Kim 2007; Harabawy and Mekkawy 2011). While researching fish’s morphology and histology in relation to seasonal changes of the Samcheon-stream, we found obvious blood vessels in *T. brevispinis* skin. Therefore, the purpose of this study was to describe and analyze the skin structure of *T. brevispinis* while focusing on cutaneous respiration.

## Materials and methods

### Specimen preparation

**Fig. 1:**
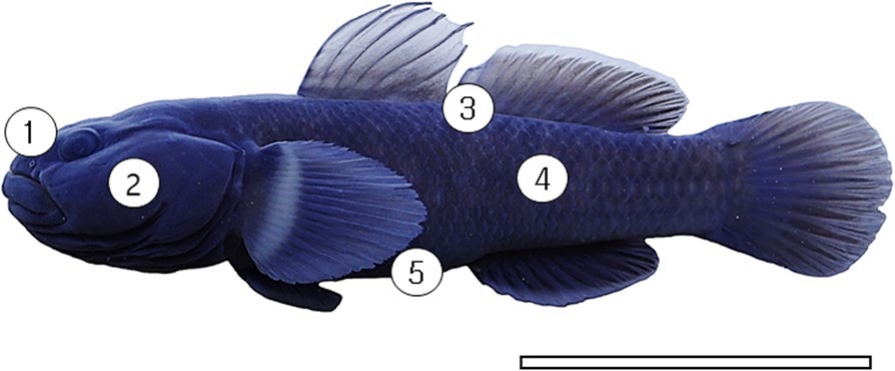
The photograph of *Tridentiger brevispinis*. The five skin regions are ①, dorsal snout; ②, operculum; ③, dorsal-caudal region; ④, lateral body; ⑤, ventral body, respectively. The bar indicates 5 cm

Eight adult *T. brevispinis* (85.2 to 11.4 mm in standard length, Fig. 1) were caught in Samcheon stream of Jungin-dong, Jeonju-si, Jeollabuk-do (35°46′59″N 127°06′33″E) using a scoop net (10 × 10 mm in nesh) in March 2022. The gobies were immediately anesthetized with 0.05% tricaine methanesulfonate (MS-222; Sigma, St Louis, MO, USA) in the field, and then fixed with 10% formalin solution buffered at pH 7.4 for 1 day. Experimental procedures obeyed the rules of Jeonbuk National University Institutional Animal Care and Use Committee for animal ethics (2016-12ET-0097).

### Microscopic investigation

Each skin sample (five regions: dorsal snout, operculum, dorsal-caudal region, lateral body, ventral body) of the eight fixed specimens was dissected from the fish’s body, respectively (Fig. 1). These fragments were immersed in an ascending series of alcohol (70%, 80%, 90%, 95%, 100%, 100%) for 1 h each, cleared in xylene, and then embedded in paraffin-wax (Oxford, paraplast) for 24 hours. The paraffin blocks were five-micrometer serially sectioned using a microtome (Leica 820, Leica Microsystems, Wetzlar, Germany), all paraffin was removed with solvent xylene, dehydrated through descending alcohol series (100%, 100%, 95%, 90%, 80%, 70%, 60%). The skin tissues were stained with hematoxylin and eosin (H&E) and Masson’s trichrome to identify the cell type and clearly identify the epidermis and dermis structures. The image on the stained tissues was obtained by a light microscope (AX10, Carl Zeiss, Germany) and analyzed with Axio vision (LE REL. 4.5, Carl Zeiss, Germany).

### Statistical analysis

SPSS statistical software (statistics version 18.0, IBM, USA) was used for statistical analysis of epidermal thickness (basement membrane to surface) and diffusion distance (blood capillary to surface) between skin regions. Kruskal-Wallis test was applied to mean comparison of epidermal thickness for non-parametric estimation (*p* < 0.05) and one-way ANOVA test for parametric estimation (*p* > 0.05) determined by Levene’s test. Pearson’s correlation coefficient was measured to identify a correlative interaction between epidermal thickness and diffusion distance.

## Results

### Histology

**Fig. 2:**
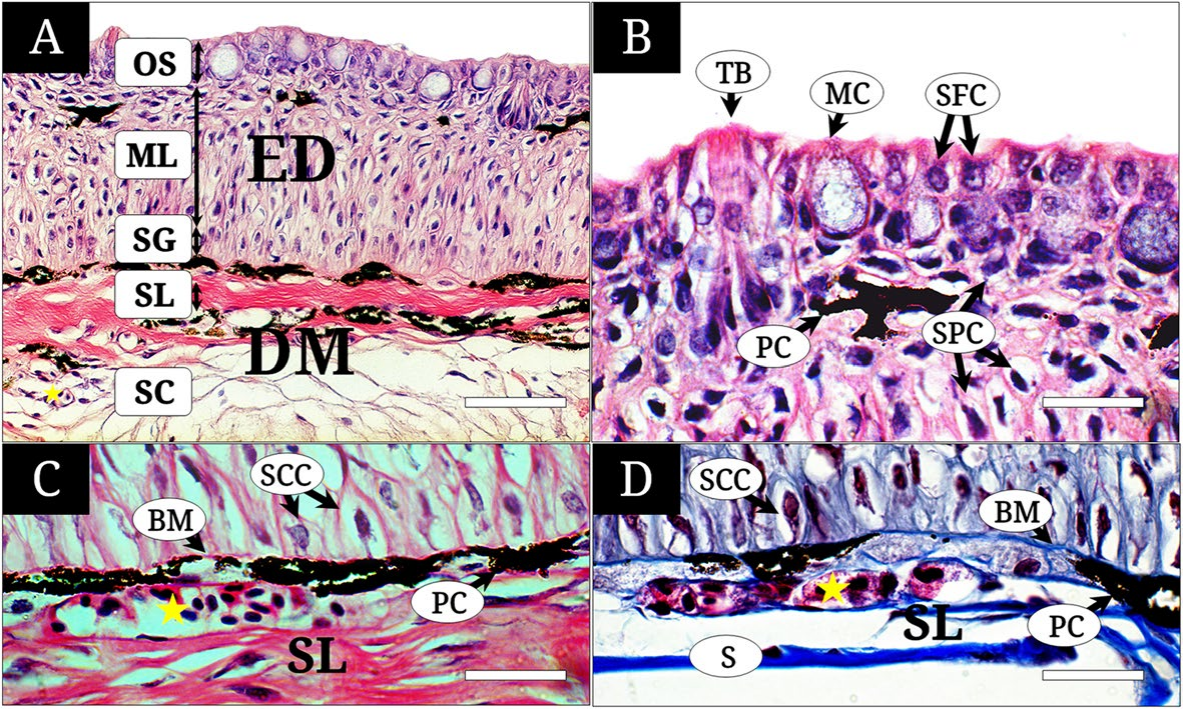
The histology of the dorsal snout and operculum skin of *Tridentiger brevispinis*, stained with hematoxylin and eosin (A, B, C) and Masson’s trichrome (D). **A** The skin (dorsal snout) structure classified largely into the epidermal (ED) with outermost surface layer (OS), middle layer (ML) and stratum germinativum (SG) and dermis (DM) with stratum laxum (SL) and stratum compactum (SC); **B** the dorsal snout consisting of mucous cells (MC), pigment cells (PC), taste buds (TB), stratified flattened cells (SFC), stratified polygonal cells (SPC); **C** and **D** the operculum showing the stratified columnar cells (SCC) of the ED above the basement membrane (BM), the blood capillaries (yellow asterisk), scale (S) and the PCs in the SL of the DM. The bars indicate 50 μm in A and 20 μm in B-D, respectively

The skin of *T. brevispinis* was classified into two large parts, the epidermis and dermis (Fig. 2A). The epidermis consisted of the outermost surface layer, middle layer, and stratum germinativum. The dermis contained the stratum laxum and stratum compactum. These five skin regions commonly contain mucous cells (MCs), pigment cells (PCs), stratified columnar cells (SCCs), stratified flattened cells (SFCs), and stratified polygonal cells (SPCs) (Fig. 2B-D). Taste buds (TB) were only observed in the dorsal snout (Fig. 2B).

**Fig. 3:**
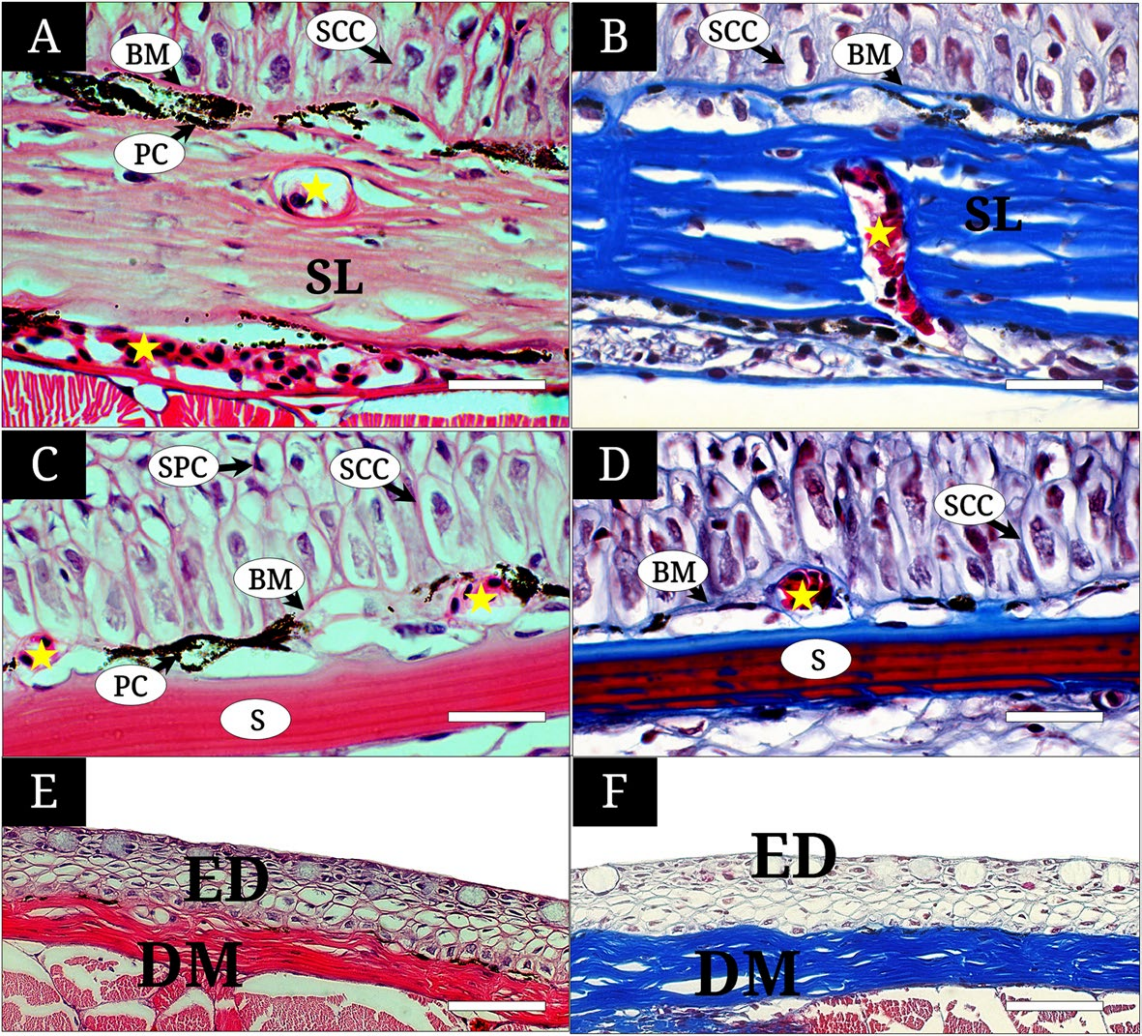
The histology of the dorsal-caudal region (A, B), lateral body (C, D), ventral body (E, F) of *Tridentiger brevispinis*, stained with hematoxylin and eosin (A, C) and Masson’s trichrome (B, D). **A** and **B** the epidermis (ED) consisting of stratified columnar cells (SCC), pigment cells (PC) distributed along the basement membrane (BM), blood capillaries (yellow asterisk) in the stratum laxum (SL); **C** and **D** the ED consisting of the SCCs, the SPCs, the PCs, and blood capillaries (yellow asterisk) and scales just below the (BM). The scale bars indicate 20 μm in A-D, and 50 μm in E-F, respectively

The MCs were large oval cell located along the outermost surface layer with a squamous nucleus at its bottom. The cytoplasm showed a faint color or were not stained with H&E (Fig. 2B). The PCs were small granule-melanophores observed both in between the outermost surface layer and middle layer, and the basement membrane and stratum laxum. PCs demonstrated a deep black color in H&E and Masson’s trichrome staining (Fig. 2A-D). The SCCs were composed of a single layer of columnar cells along the basement membrane. SCCs showed an oval nucleus with violet color upon staining with H&E and purple color with Masson’s trichrome (Figs. 2C and D, 3A-D). The SFCs were squamous or cuboidal cells with reduced cytoplasm of pink color stained more deeply than the SPCs in H&E (Fig. 2B). SPCs had a polygonal shape, unregular cytoplasm, and made up five to ten layers of the middle layer. They had a weak pink cytoplasm on H&E staining. The taste bud was a neuron bundle with a long dendrite extending to the surface. Its nucleus was located at the basal layer of the outermost surface layer (Fig. 2B).

Well-developed dermal vascularization was confirmed in the connective tissue of the three skin regions (operculum, dorsal-caudal region, lateral body) (Figs. 2C-D, 3A-D) but little in the dorsal snout (Fig. 2A) and absent in the ventral body (Fig. 3E-F). Some blood capillaries wrapped with dermal collagens of the dermis were only observed just below the basement membrane. Occasionally, they protruded slightly into the epidermis of the lateral body (Fig. 3C and D).

### Morphometry

**Fig. 4:**
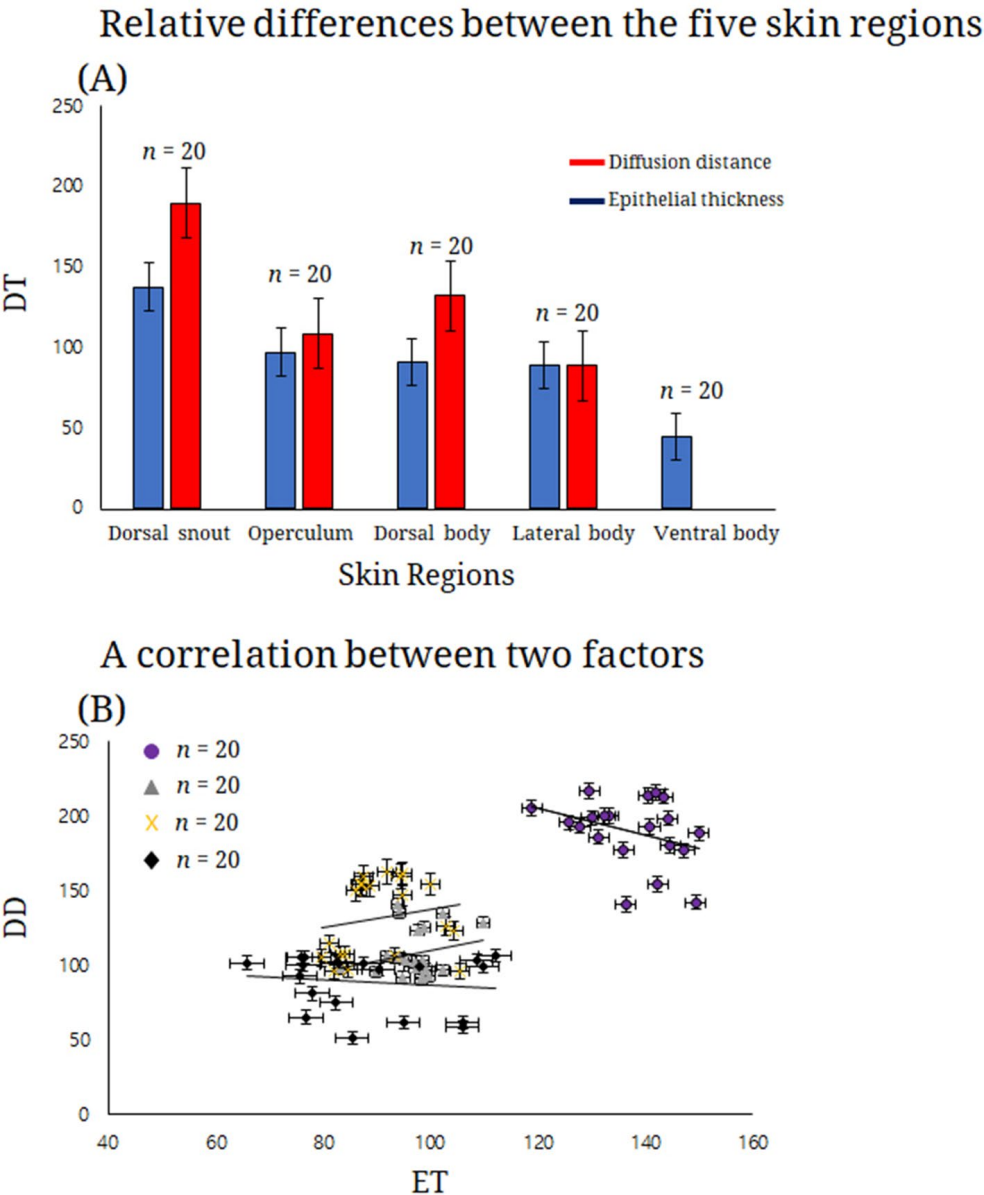
Relative differences (**A**) in the epidermal thickness and the diffusion distance on the five skin regions; A correlation (**B**) between two factors, epidermal thickness (x-axis, *n* = 20) and diffusion distance (y-axis, *n* = 20). Circle, dorsal snout; triangle, operculum; x, dorsal-caudal region; diamond, lateral body. ET, epithelial thickness; DD, diffusion distance; DT, diffusion distance and epithelial thickness values

The epidermal thickness demonstrated regional differences: the dorsal snout showed the highest value (137.3 ± 8.5 μm, 118.9–150.0; mean ± SD, range), followed by the operculum (96.9 ± 5.3 μm, 83.3–109.9), dorsal-caudal region (90.8 ± 7.9 μm, 79.7–105.6), lateral body (88.6 ± 13.8 μm, 65.9–112.1), while the ventral body was the lowest (44.2 ± 5.3 μm, 35.4–52.4). These values showed a highly significant difference in epidermal thickness (Kruskal-Wallis test, χ^2^ = 78.944, *df* = 4, *p* < 0.001; Fig. 4A). In addition, the diffusion distance showed regional differences in the minimum distance between blood capillary and the skin surface. The dorsal snout has the highest (189.7 ± 22.4 μm, 141.2–216.9; mean ± SD, range) followed by the dorsal-caudal region (131.9 ± 25.9 μm, 95.7–163.0), operculum (108.3 ± 16.7 μm, 90.7–141.4), and lateral body (88.6 ± 19.0 μm, 51.4–106.7). These values demonstrated a highly significant difference in diffusion distance measurement (one-way ANOVA, *df* = 3, *f* = 8.835, *p* < 0.001; Fig. 4A). In total, the two parameters (epidermal thickness and diffusion distance) were highly positively correlation in the four skin regions (dorsal snout, operculum, dorsal-caudal region, and lateral body) (Pearson’s correlation coefficient, *r* = 0.719, *p* < 0.001; Fig. 4B).

## Discussion

The fish skin is a body integument that provides diverse barriers and chemical passages as follows: i) the maintenance of water- and ion-osmotic balance (Ghioni et al. 1997), ii) a physical barrier to prevent water loss (Sayer 2005) and entry of harmful substances (Shephard 1993) or potential infective pathogens (Benhamed et al. 2014), iii) outer region for color expression (Zarnescu 2007), iv) sensory reception of physical and chemical stimuli (Bleckmann and Zelick 2009), and v) cutaneous respiration in the case of some teleosts (Urbina et al. 2014). Considering the above reports, the skin of *T. brevispinis* contained SFCs, SPCs, SCCs, MCs, PCs, and TBs that may enable the maintenance of a stable skin structure (Roberts and Horsley 2014), improve tolerance to somewhat turbid water with high amounts of organic materials (Han and An 2013; Park and Gwak 2019), perceive chemical differences between sour, salty, sweet, and bitter foodstuffs, and adjust to environmental changes in its habitat (Morais 2017). *Tridentiger brevispinis* also shows significant characteristics for cutaneous respiration (Glover et al. 2013): 1) a thick epidermis (91.6 ± 31.0 μm, 35.4–150.0), 2) well-developed dermal vascularization (in the operculum, dorsal-caudal region, and lateral body), and 3) some blood capillaries with dermal collagen protruding slightly into the epidermis (only in the lateral body).

Underwater teleosts or amphibious species have large cells (club cells, mucous cells, swollen cells) that play a major role in absorbing dissolved gas in water and spreading supplemental oxygen to the blood capillary or connective tissue (Park et al. 2001; Lauriano et al. 2018). Among them, Korean underwater species that inhabit stream regions where water level variation happens frequently (Kim and Park 2002) commonly have a thicker epidermis as follows: 53.2–111.7 μm in the freshwater goby *Rhinogobius brunneus* (unpublished) with numerous MCs, 97.5–113.5 μm in the Chinese muddy loach *Misgurnus mizolepis* (Park et al. 2001), 87.8–137.1 μm in the Korean spined loach *Iksookimia koreensis* (Park, 2002a, b) with numerous MCs and club cells, and 59–297.0 μm in the Korean eel goby *Odontamblyopus lacepedii* (Park et al., 2003b) with abundant MCs and swollen cells. *Tridentiger brevispinis* has a thicker epidermis as well that is augmented by multi-layered the SPCs and the possession of abundant MCs to promote oxygen diffusion at about 70% of the absorption rate in water (Ultsch and Gross 1979). In addition, because a reduced diffusion distance facilitates a higher diffusion velocity of supplementary oxygen, a short length between the blood capillaries and skin surface in many teleosts has been suggested to facilitate cutaneous respiration (Kim and Park 2011; Glover et al. 2013). So, the reduced diffusion distance of the lateral body in *T. brevispinis* may be considered the most efficient spot for cutaneous respiration among the five regions studied.

In skin vascularization, highly dermal blood vessels are a histological modification for oxygen-carbon dioxide exchange in fish skin (Potter et al. 1995; Welsch and Potter 1998). In addition, intraepidermal blood capillaries or dermal capillaries near the epidermis allow fish more efficient oxygen absorption than those in the dermis due to the reduced distance between external gas and the blood capillary (Park et al. 2003a; Park and Kim 2007). So, teleosts living in poorly oxygenated water have been reported to exhibit thicker and wider vascularization and a well-developed dermal papillae of blood capillaries positioned closer to the epidermis. In the lungfish, *Neoceratodus forsteri*, which is extremely well-adapted to the aerial exposure, abundant blood vessels supplying the papilla and subepidermal capillary network occurs among the fibrous layer of the dermis (Bemis and Northcutt 1992). Blood capillaries distributed just below the basement membrane were revealed in *M. mizolepis* (Park et al. 2001) and *I. koreensis* (Park 2002b). In addition to such positions of the stratum laxum, the Korean torrent catfish *Liobagrus mediadiposalis* (Park et al. 2003a) and the Korean stumpy bullhead *Pseudobagrus brevicorpus* (Park and Kim 2007) showed blood capillaries in the middle part of the epidermis. All of these examples are best suited to survival in a hypoxic wetland or pool of freshwater prone to drying due to the Korean climate (Kim and Park 2002). *Tridentiger brevispinis* has fine blood capillaries just below the basement membrane protruding into the epidermis and visible only in the lateral body. With this modification, the diffusion distance between blood capillary and surface in *T. brevispinis* is lowest in the lateral body (88.6 ± 19.0 μm), followed by the operculum (108.3 ± 16.7 μm). This suggests that the skin region of the lateral body of *T. brevispinis* is the most effective spot for cutaneous respiration. Moreover, well-developed vascularization and blood capillaries near the epidermis in *T. brevispinis* is a skin modification suitable for obtaining dissolved gas under hypoxic water conditions.

Consequently, *T. brevispinis* exhibits a thicker epidermis, a well-developed vascularization, a few blood capillaries protruding into the epidermis, and reduced diffusion distance in the lateral body, all of which are related to effective cutaneous respiration for survival in a hypoxic habitat during the dry season.

## Conclusions

The Korean trident goby *Tridentiger brevispinis* showed a thicker epidermis having taste buds, stratified flattened cells, stratified polygonal cells, mucous cells, stratified columnar cells, and pigment cells. Remarkably, the dermis showed well-developed dermal vascularization (in the operculum, dorsal-caudal region, and lateral body) and a few blood capillaries (in lateral body) just above the basement membrane, except for the ventral body (not confirmed). The epidermis thickness was the highest value (137.3 ± 8.5 μm, 118.9–150.0; Mean ± SD, Range) and the ventral body was the lowest (44.2 ± 5.3 μm, 35.4–52.4) (Kruskal-Wallis test, χ^2^ = 78.944, *df* = 4, *p* < 0.001). The diffusion distance was more reduced in the lateral body than other regions (one-way ANOVA, *df* = 3, *f* = 8.835, *p* < 0.001). Epithelial thickness and diffusion distance have a highly significant correlation (Pearson’s correlation coefficient, *r* = 0.719, *p* < 0.001). These results demonstrate the goby’s adaptation to cutaneous respiration to overcome hypoxic conditions during periodic dry periods.

## Data Availability

Not applicable.

## Abbreviations

MC: Mucous cell
PC: Pigment cell
SCC: Stratified columnar cell
SFC: Stratified flattened cell
SPC: Stratified polygonal cell
TB: Taste bud
